# Natural vs. genetically engineered microbiomes: understanding public attitudes for indoor applications and pathways for future engagement

**DOI:** 10.3389/fgene.2025.1560601

**Published:** 2025-03-26

**Authors:** Christopher L. Cummings, Kristen D. Landreville, Jennifer Kuzma

**Affiliations:** ^1^ Genetic Engineering and Society Center, Raleigh, NC, United States; ^2^ Engineer Research and Development Center (ERDC), Vicksburg, MS, United States

**Keywords:** microbiome engineering, genetic engineering, public perceptions, responsible research and innovation (RRI), built environment

## Abstract

This study examines public preferences for natural microbiomes and support for genetically engineered (GE) microbiomes in the built environment, focusing on the demographic, sociographic, and attitudinal factors that influence these preferences. Using data from a nationally representative survey of 1,000 U.S. adults, we employed hierarchical regression analyses to assess the relative contribution of these variables. While demographic and sociographic factors explained limited variance, topic-specific attitudes, including positive perceptions of microbiome engineering’s potential to improve quality of life, were the most significant predictors of support. Conversely, age, distrust in science, and perceived knowledge negatively influenced support for GE microbiomes, reflecting skepticism among some audiences. The findings highlight the potential of the Responsible Research and Innovation (RRI) framework to align the development of microbiome engineering with societal values and to address diverse public perspectives. This research provides actionable insights for policymakers, researchers, and communicators seeking to navigate the complexities of public engagement with emerging biotechnologies.

## Introduction

In recent years, a nascent yet rapidly evolving field, precision microbiome engineering, has evolved at the intersection of microbiology, genomics, and engineering. This field focuses on exploring opportunities for purposeful manipulation of microbial communities inhabiting a variety of environments in order to explore the potential of shaping microbiomes to promote public health, environmental sustainability, and overall wellbeing (Liang et al., 2019; Parker and Kunjapur, 2020). The ability to precisely engineer microbiomes holds great potential for creating targeted therapies and improving our understanding of the complex interactions between microorganisms and their environment (Langdon et al., 2016). One subset of microbiome engineering aims to intentionally design, alter, or enhance microbial communities to address a variety of challenges within the built environment (BE). The BE encompasses all homes, workplaces, schools, and other indoor spaces where the average person can spend upward 90% of their life. The BE contains diverse microbial populations (bacteria, fungi, viruses, etc.) with which people interact via air circulation, water flowing through plumbing, and surface contact (Li et al., 2021; Klepeis et al., 2001; Adams et al., 2016; Gilbert and Stephens, 2018; Leung and Lee, 2016).

Microbial communities within the BE are commonly thought to cause or exacerbate, and also prevent or mitigate, human disease through exposure pathways (inhalation, transdermal contact, ingestion, etc.). Microbiomes in the BE are influenced by factors like sunlight, ventilation, temperature, moisture, building materials, and plumbing systems (Li et al., 2021). Technologies addressing these factors include antimicrobial materials, advanced HVAC systems, sunlight-optimized designs, pathogen-reducing plumbing, UV disinfection, and the introduction of beneficial microbes via indoor plants (Li et al., 2021). However, such strategies must account for the risk of pathogens evolving resistance (Graves, 2021).

Microbiome engineering of the built environment (MEoBE) therefore seeks to improve inhabited spaces ranging in technological interventions from improving air quality to mitigating the spread of pathogens (Parker and Kunjapur, 2020). Emerging strategies include integrating probiotic bacteria into air systems to suppress airborne pathogens, utilizing UV radiation for microbial control, and optimizing ventilation to promote healthier microbial ecosystems (Dai et al., 2017; Gilbert and Stephens, 2018). Natural microbiomes, such as those derived from soil bacteria or indoor plants, are being explored for their ability to introduce beneficial microbes into indoor spaces, promoting a balance that deters harmful pathogens while supporting occupant health (Gilbert and Stephens, 2018; Gilbert and Hartmann, 2024; Leung and Lee, 2016). In contrast, genetically engineered (GE) microbiomes, such as those designed to degrade volatile organic compounds or produce antimicrobial substances, offer targeted solutions to indoor environmental challenges, such as pollution reduction and disease prevention (e.g., Tao et al., 2022; Parker and Kunjapur, 2020). Innovative materials such as antimicrobial surfaces and advanced HVAC designs aim to minimize harmful microbial exposure while supporting beneficial microbiomes (Leung and Lee, 2016). These interventions highlight the dual potential of MEoBE to address public health needs and reshape indoor microbial ecosystems. However, these approaches must be carefully evaluated to ensure that interventions do not inadvertently encourage the evolution of microbial resistance or disrupt ecological balance (Graves, 2021; Mathur and Roy, 2024).

As promising as this field may seem, its advancement must be coupled with careful consideration of societal perspectives, values, and ethical concerns (Hardwick et al., 2024). At the heart of this emerging field lies a critical question: How should researchers and developers of MEoBE technologies prioritize the input and desires of the public? As MEoBE continues to develop, its applications will directly impact people’s lives in intimate, indoor settings, making it essential to align technological innovation with the expectations, understanding, and comfort of the communities it seeks to benefit. In this research study, we advocate that the Responsible Research and Innovation (RRI) framework is best suited to achieve this alignment which emphasizes inclusivity, reflexivity, transparency, and responsiveness in guiding technological development (Stilgoe et al., 2013). RRI underscores the importance of embedding public attitudes and values into the research process, ensuring that biotechnology advancements like MEoBE are not only innovative but also socially desirable, ethically acceptable, and aligned with public will (Trump et al., 2023).

Despite the promising implications of MEoBE, to our knowledge, only one prior study, (co-authored by the authors of this article), has sought to comprehensively and representatively survey public opinion in the United States on this topic. In this prior study, we found that certain general cultural and demographic factors influenced general attitudes about microbiome engineering, such as age, political affiliation, education level, and trust in science (Cummings et al., 2024). Also, almost half of respondents across demographic groups were uncertain about the benefits and risks of technology, and a majority felt that government oversight was important to ensure ethical and responsible development. However, in that study, we did not compare which factors specifically influenced attitudes towards genetically engineered microbiomes used for MEoBE *versus* naturally-occurring microbiomes used for MEoBE. While some research has explored general attitudes toward microbiome science, genetic engineering, and a host of biotechnologies like genetically modified organisms and gene-edited foods (e.g., Yeo et al., 2019; Kokkinias et al., 2024; Cummings and Peters, 2022; Ewa, 2022; Hallman, 2002; Mehta, 2001), there remains a notable gap in understanding how individuals perceive both natural microbiomes and genetically engineered (GE) microbiomes specifically designed for indoor spaces. This gap is significant, as public preferences and concerns will likely shape the trajectory of MEoBE technologies and influence their eventual adoption.

For the purposes of this study, we define natural microbiomes as microbial communities introduced into the built environment without intentional genetic modification and genetically engineered (GE) microbiomes as those that have been altered through genetic modification to achieve specific functions. These distinctions were also briefly explained to participants within the survey to support consistent interpretation. The purpose of this study, therefore, is to address this critical gap by systematically examining public attitudes and preferences toward natural and GE microbiomes in the built environment. Furthermore, this research is part of a broader initiative connected to an active National Science Foundation (NSF) Engineering Research Center (ERC) focused on the development and deployment of engineered microbiomes in the built environment. While microbiome engineering technologies remain in their early stages, the fact that research and development efforts are already underway underscores the urgency of assessing public attitudes before these innovations are widely implemented. Early assessment of downstream public perceptions is essential to inform proactive communication, engagement strategies, and even to shape technological trajectories in ways that align with public values and ethical concerns. Despite this urgency, no prior research has systematically examined public preferences regarding natural *versus* genetically engineered microbiomes in indoor spaces. This gap, alongside the concurrent advancement of the technology, provides a timely and compelling justification for the present study. By surfacing public concerns and expectations early in the innovation process, this work aims to contribute to more socially responsive and ethically informed pathways for microbiome engineering in the built environment. By employing a representative survey of the U.S. population, this research seeks to identify the demographic, sociographic, and attitudinal factors that shape these preferences. In doing so, we discuss how our findings can provide actionable insights for researchers, policymakers, and developers seeking to engage with diverse communities through the lens of the RRI framework. By linking public attitudes with responsible innovation strategies, this study aspires to foster an inclusive dialogue that aligns the development of microbiome engineering technologies with the values and expectations of society. From this premise, we articulate the following research questions that guide this inquiry, “What are the key individual factors that differentiate public preferences for natural microbiomes from support for genetically engineered (GE) microbiomes? How can these insights inform the development of inclusive, ethical, and socially responsive microbiome engineering strategies for indoor spaces?”

This research therefore aims to inform the development of microbiome engineering strategies that are inclusive, ethical, and reflective of societal values, ensuring that technological advancements are guided by a comprehensive understanding of public attitudes. What follows is a review of RRI and biotechnology perception studies followed by a detailed explication of our methods, results, and discussion.

## Literature review

### Responsible Research and Innovation

The framework of RRI was introduced in the early 2010s by scholars in science and technology studies, particularly in Europe, as a way to broaden the scope of governance for emerging technologies beyond risk assessment to include societal values, ethical considerations, and inclusive public engagement (Stilgoe et al., 2013). Unlike traditional risk governance approaches such as cost-benefit analysis, technology assessment, or regulatory science that primarily address downstream risks of technological products, RRI emphasizes upstream considerations—such as public values, motivations for innovation, and inclusive deliberation—throughout the research and development process (Stilgoe et al., 2013). Stilgoe et al. (2013) argue that public controversies over science and technology extend beyond questions of risk, encompassing broader societal concerns about the objectives and values driving research. RRI therefore aims to align scientific and technological development with democratic processes, societal values, and a forward-looking humility toward unforeseen consequences (Owen et al., 2012; Owen et al., 2013; Stilgoe et al., 2013).

Rooted in a longer tradition of work on the ethical, legal, and social implications/aspects (ELSI in the United States or ELSA in the EU) of science and technology, RRI has gained traction in the EU, where it has been integrated into funding programs and studied extensively in science and technology studies (STS) scholarship (Felt, 2018). However, RRI has not been widely adopted in the United States within funding mechanisms, research policies, or innovation systems. Despite this, RRI principles offer a promising framework for biotechnology developers seeking to enhance public engagement and legitimacy in their work. The most cited article on RRI identifies four core principles: anticipation, inclusion, reflexivity, and responsiveness (Stilgoe et al., 2013). *Reflexivity* encourages researchers to move beyond purely risk-based governance to reflect on the underlying goals, assumptions, limits of knowledge, and alternative framings of research problems. *Anticipation* focuses on analyzing and exploring potential future consequences before technological development begins, improving the capacity to address downstream risks. *Inclusion* emphasizes broadening governance by incorporating diverse public perspectives rather than limiting input to subject-matter experts, fostering a richer, more reflexive approach. Finally, *responsiveness* entails the ability to adapt in response to new data, changing circumstances, or emerging impacted groups and public values. Together, these four principles form the foundation of what Wittrock et al. (2021) describe as a “more responsible vision of innovation” compared to other frameworks focused on research ethics or STEM diversity. RRI has been operationalized by national funding bodies and integrated into research practices in the EU (Wittrock et al., 2021), suggesting a pathway for its broader adoption in shaping more inclusive and democratically aligned biotechnology development. In this study, we employ the RRI framework not only as a conceptual lens but also as an analytical guide to inform the selection of variables in our regression models. For instance, variables related to inclusion (e.g., demographic diversity), reflexivity (e.g., perceived knowledge), and responsiveness (e.g., trust in science and attitudes toward improving society) are grounded in the four core RRI principles. By embedding these elements into our model design, we aim to assess how these value-laden factors correlate with public preferences for microbiome technologies, consistent with RRI’s goal of aligning innovation with societal values.

### Perceptions of microbes and microbiome engineering

The public’s perceptions of microbes and microbiome engineering are complex, shaped by both knowledge gaps and emotional responses. For instance, Kokkinias et al. (2024) conducted semi-structured interviews and surveys with 30 participants, revealing that while microbes are commonly associated with disease, many participants also recognized their beneficial roles in health and ecological systems. However, confusion about microbial terminology, such as the distinction between bacteria and viruses, persisted and the author’s note that negative perceptions significantly influenced participants' willingness to engage with microbial topics, underscoring the impact of emotions on public attitudes. In a similar example, Yeo et al. (2019) specifically explored the influence of disgust on public perceptions of microbiome modification through their perception study in response to introducing the process of fecal microbiota transplants for treating severe *Clostridium difficile* infections. Their experimental survey found that disgust-inducing language heightened perceived risks, particularly when content focused on humans. These findings emphasize how negative emotional reactions may shape public attitudes toward the acceptability of microbiome technologies—at least for this instance of fecal transplant.

Robinson et al. (2021) further explicated the roles of knowledge, attitudes, and nature engagement in shaping perceptions of microbes. Their study showed that greater knowledge of microbes, such as correctly identifying archaea and protozoa, correlated with more positive attitudes. However, identifying viruses as microbes often led to more negative views, likely influenced by the COVID-19 pandemic. Additionally, spending more time in natural environments was associated with more favorable attitudes, suggesting that nature engagement may reduce negative perceptions of microbes. Importantly, perceived knowledge—defined as one’s self-assessed understanding—can diverge from objective knowledge and may influence attitudes in complex ways. Light et al. (2022) found that individuals who overestimated their scientific knowledge were more likely to reject expert consensus on issues like GMOs and vaccines. In our study, we distinguish between perceived familiarity (awareness of the concept) and perceived knowledge (confidence in understanding), a distinction explicated in the Methods section, which helps capture these different dimensions of self-assessment and their effects on support for microbiome engineering.

Perceptions among students similarly highlight the dominance of negative views. Karadon and Şahin (2010) found that over 50% of school-aged children described microorganisms as “dirt,” “pollutant,” or “harmful,” and most associated microbes with risks rather than benefits. Jones et al. (2012) observed that college students' knowledge of microbes and microbial transmission improved after taking an undergraduate microbiology class, with a deeper understanding of microbes’ ecological roles. However, this knowledge did not necessarily translate to changes in health-related behaviors, such as vaccine uptake, indicating that microbial transmission knowledge did not impact specific decisions about health protection.

Broader public understanding also reflects these trends. A kiosk-based survey conducted at the American Museum of Natural History in New York City from 2016 to 2018 gathered responses from over 22,000 visitors from 172 countries and territories (Zichello et al., 2021). The survey found that only 50% of respondents could correctly identify penicillin as an antibiotic, and fewer than 50% viewed microbes as beneficial. Zichello et al. (2021) argue that this lack of understanding likely impedes acceptance of public health measures that rely on the demonstrated benefits of microbes, a concern corroborated by Thaler et al. (2019). Finally, Shan et al. (2019) stress the importance of clear, transparent communication from microbiome researchers. Overstating the efficacy or significance of findings can mislead the public and press, potentially undermining trust in the scientific community. Responsible communication is essential for fostering a balanced understanding of microbiome science and its applications.

### Perceptions of other biotechnologies

The field of microbiome engineering is still in its early stages, with few studies examining public perceptions. To better contextualize this work within the broader biotechnology landscape and understand how similar variables may shape attitudes toward microbiome engineering in the BE, we also review some studies about public attitudes toward more established biotechnologies, such as genetically modified organisms (GMOs) and gene-edited foods.

Demographic and sociographic characteristics have been reported as key predictors of public perceptions toward various biotechnologies. For instance, research consistently finds that men are more supportive of gene-editing technologies than women, while younger individuals exhibit greater acceptance compared to older age groups (Critchley, 2018; Mehta, 2001). Education level presents mixed effects: some studies report that higher education correlates with increased support for biotechnologies, while others find no significant relationship (Ewa, 2022). Religiosity also plays a role, with individuals reporting lower levels of religiosity generally expressing greater acceptance of gene-editing applications (Cummings and Peters, 2022). Regarding gender, women, even after controlling for knowledge and trust, tend to express more concern about applications involving humans, such as embryonic editing (Critchley, 2018). Ethnicity has also been reported to shape perceptions, with varying levels of unease depending on cultural and regional contexts (Mehta, 2001). For instance, Australians have been shown to express less concern about human embryo editing than non-Australians but are more apprehensive about animal applications (Ewa, 2022). Socioeconomic status influences the willingness to adopt gene-edited foods, with individuals from lower-income backgrounds exhibiting lower acceptance rates (Cummings and Peters, 2022). Political ideology shows mixed effects on perceptions of GMOs and gene-edited foods. Some studies indicate that individuals with left-wing political orientations are more supportive of biotechnologies, reflecting a focus on scientific progress and innovation (Critchley, 2018). However, other research finds no consistent relationship between political ideology and attitudes toward these technologies (Mehta, 2001). Ideological factors appear to be less predictive of public attitudes than moral and ethical considerations, which often have a stronger influence on perceptions of biotechnologies (Kato-Nitta et al., 2021).

Religious beliefs have also been demonstrated to significantly impact public acceptance of biotechnologies. Higher religiosity is consistently associated with lower support for gene-editing technologies (Critchley, 2018; Mehta, 2001). Religious individuals often raise concerns about “playing God” or transgressing natural boundaries, especially in contexts involving humans or animals (Hallman, 2002). These objections are often framed around moral and ethical issues, with many religious respondents perceiving such interventions as violating fundamental values (Cummings and Peters, 2022). Trust/distrust in science as a means for solving societal problems has also been demonstrated to influence public acceptance of biotechnologies, with individuals who trust science and scientists being more likely to view GMOs and gene-edited foods favorably (Cummings and Peters, 2022; Thaler et al., 2019). These findings highlight the complexity of public perceptions and the need for nuanced approaches to communication and engagement with diverse audiences. Together these factors inform our inquiry as detailed in the methods section below.

## Methods

### Sampling procedure

This study employed a cross-sectional survey to assess public attitudes toward microbiome engineering in the built environment. Data were collected from a nationally representative sample of 1,000 U.S. residents aged 18 and older, drawn from YouGov’s National Omnibus Panel during the first 2 weeks of December 2023. A total of 1,092 respondents completed the survey, and the dataset was matched down to 1,000 respondents to align with a sampling frame based on gender, age, race, and education. The sampling frame, a politically representative “modeled frame” of U.S. adults, was constructed using data from multiple sources, including the American Community Survey (ACS) public use microdata file, public voter records, the 2020 Current Population Survey (CPS) Voting and Registration supplements, the 2020 National Election Pool (NEP) exit poll, and the 2020 Cooperative Election Study (CES) surveys. This comprehensive approach ensured the inclusion of demographic characteristics and 2020 presidential voting data. The survey results have an observed margin of error of ±3.38 percentage points. YouGov’s National Omnibus Panel, comprising 1.8 million U.S. residents, recruits participants through diverse methods to enhance representativeness. These include web advertising, permission-based email contacts, partner-sponsored solicitations, telephone calls via random digit dialing, and mail outreach using random address selection (YouGov, 2020). While this study utilized an opt-in panel with demographic weighting to approximate the representativeness of the U.S. population, it is important to acknowledge the small potential for selection bias despite these efforts.

While a sample of 1,000 respondents may seem modest relative to the overall U.S. population, this sample size is standard for national opinion surveys and is sufficient to achieve a margin of error of approximately ± 3.38 percentage points at a 95% confidence level. This allows for statistically meaningful generalizations to the broader population when combined with careful stratified sampling and demographic weighting procedures. Nonetheless, we acknowledge that any survey, regardless of sample size, may be subject to sampling and non-sampling errors. Individuals who voluntarily participate in online surveys may differ in ways not fully captured by demographic matching, such as potentially being more engaged with digital platforms or having stronger interest in scientific topics. We acknowledge this potential limitation and encourage cautious interpretation of findings accordingly.

### Sample characteristics

The average age of respondents was approximately 49 years (SD = 17.71), ranging from 19 to 88 years. The gender distribution included 480 men, 510 women, and 10 respondents who did not select either man or woman. Approximately one-third of respondents (n = 346) reported earning a 4-year college degree or higher, while the remaining two-thirds (n = 654) had not completed a 4-year degree. Income levels were distributed across 267 lower-income respondents (earning less than $30,000 per year), 443 middle-income respondents ($30,000 to $99,999 per year), and 290 higher-income respondents (earning more than $100,000 per year). The sample included respondents from diverse racial backgrounds: 64.7% identified as White, 12.1% as Black or African American, 14.8% as Hispanic or Latino, 3.3% as Asian, and 5.1% as Native American, Middle Eastern, or other. Respondents’ population density per square mile was calculated using their zip code and ranged from 3 to 125,860 people, with an average of 5,185 people and median of 1,425 people. Metro area status was also assessed using zip code, with 85.1% of respondents living within a metropolitan area (i.e., central counties with one or more urban areas with populations of 50,000 or more people) and 14.8% in non-metro regions (areas with less than 50,000 people). Table 1 shows key demographic comparisons between our study sample and the U.S. population (US Census Bureau, 2025).

**TABLE 1:** Comparison of sample demographics and United States census demographics.

	Sample	Census estimate
Age (65 years and older)	23.3%	17.7%
Sex (Female)	51%	50.5%
Race (White, not Hispanic)	64.7%	58.4%
Education (4-year college degree or higher)	34.6%	35.0%
Median household income range	$50,000-$59,000	$70,000-$79,000

### Independent variables

In addition to demographic and sociographic variables, the study incorporated independent variables identified in prior research as predictors of preferences for natural microbiomes and support for genetically engineered (GE) microbiomes in the built environment. Political orientation was measured using a seven-point scale of party identification (strong Republican coded high and strong Democrat coded low), with a mean score of 3.82 (SD = 2.21), indicating a moderate distribution across general political views. Religiosity was assessed with church attendance, ranging from 1 (rarely attends) to 6 (attends weekly), with a mean of 2.78 (SD = 1.77).

Two subscales measuring science and technology beliefs were also included (Rosenthal and Cummings, 2021; Cummings et al., 2024). Scale response options reported here and in the following paragraphs ranged from 1 (strongly disagree) to 5 (strongly agree). Four items comprised of the ‘distrust in science/scientists’ subscale (e.g., “Scientists do not value my concerns when making decisions; ” *M* = 2.96, *SD* = 0.87, Cronbach’s α = 0.77) and three items comprised the ‘science improves society’ subscale (e.g., “Our leaders should use technology to solve problems in society; ” *M* = 3.72, *SD* = 0.74, Cronbach’s α = 0.69). See the Supplementary Material for the full-scale items.

Familiarity with microbiome engineering was assessed through composite responses to multiple items (e.g., “I am familiar with the concept of microbiome engineering; ” *M* = 2.57, *SD* = 0.96, Cronbach’s α = 0.83) and indicated low to moderate familiarity. Perceived knowledge of microbiome engineering, measured via self-reported understanding (e.g., “I have a basic understanding of how microbiome engineering can be applied; ” *M* = 2.71, *SD* = 0.92, Cronbach’s α = 0.84) also reflected moderate levels of self-reported knowledge.

Last, positive attitudes toward microbiome engineering were assessed through ten items evaluating its perceived influence on quality of life (e.g., “I believe that microbiome engineering can play a significant role in addressing environmental challenges; ” *M* = 3.26, *SD* = 0.82, Cronbach’s α = 0.96). The composite mean score indicated generally favorable attitudes toward microbiome engineering. Together, these variables provided a robust framework for examining the factors shaping public preferences for natural microbiomes and support for GE microbiomes in the built environment.

### Dependent variables

Prior to measuring dependent variables, participants were provided with a brief description distinguishing natural and genetically engineered microbiomes to help ensure shared understanding. The survey text explained: “Within the field of microbiome engineering, scientists are exploring different types of microbiomes that may help indoor spaces become healthier. Some of the microbes in these microbiomes would be unchanged by scientists as they occur in nature. Other microbes would be changed by scientists through genetic modification; these are called ‘genetically engineered microbiomes.” This framing offered a basic operational distinction that supported comprehension while minimizing technical jargon. While lay interpretations may still vary, this language ensured a baseline understanding consistent with the study’s conceptual framework. To answer our research questions, we created two dependent variables. The first, preference for natural microbiomes over genetically engineered microbiomes, was measured using the item, “I am more comfortable with naturally occurring microbiomes in indoor spaces than genetically engineered microbiomes” (*M* = 3.68, *SD* = 0.91). The second dependent variable, *support for genetically engineered microbiomes,* used the item, “I am open to the idea of using genetically engineered microbiomes if their benefits are well-documented and their risks are considered” (*M* = 3.43, *SD* = 1.06).

### Analytic approach

We used ordinary least squares hierarchical regression analyses to test our research questions. Variables in the two regression models were entered in blocks based on their presumed causal order. In the first block, control variables (demographic factors) such as age, gender, race, population density, and metro area status were included. Sociographic variables, including the highest level of education and annual income, were entered in the second block. The third block contained general value predispositions, specifically political orientation and church attendance, while the fourth block included science-specific value predispositions, such as distrust in science and positive beliefs about the societal benefits of science. The fifth block focused on familiarity with microbiome engineering, perceived knowledge, and attitudes toward microbiome engineering.

## Results

The first block included the demographic variables age, gender, race, population density, and metro area status. In Model 1, none of these variables showed significant associations, explaining only 0.7% of the variance. In Model 2, however, age emerged as a significant predictor (β = −0.176, p < 0.001), indicating that older individuals were less likely to support GE microbiomes. The demographic block contributed 4.0% of the variance (p < 0.001) in Model 2.

Sociographic variables, including education level and annual income, were added in the second block. In Model 1, neither educational level nor annual income was significant. However, in Model 2, education exhibited a positive association with support for GE microbiomes (β = 0.119, p < 0.001). Annual income had no significant effects on Model 2. The sociographic block explained 0.7% (ns) of the variance in Model 1% and 3.5% (p < 0.001) in Model 2.

The third block included general value predispositions, specifically political orientation and church attendance. Political orientation was not significant in Model 1 (β = 0.024, p = 0.474), showing no relationship to preference for natural microbiomes. But it was significant in Model 2, such that Republican Party orientation was associated with lower openness to GE microbiomes (β = −0.232, p < 0.001). Church attendance was a significant positive predictor in Model 1 (β = 0.123, p < 0.001), suggesting that individuals who attended religious services more frequently were more likely to prefer natural microbiomes. However, church attendance was not significant in Model 2 (β = 0.006, p = 0.845), suggesting that church attendance is not related to openness to GE microbiomes. These general value predispositions accounted for 1.6% (p < 0.001) of the variance in Model 1% and 5.1% (p < 0.001) in Model 2.

The fourth block focused on science-specific value predispositions, including distrust in science and beliefs about science’s societal benefits. Distrust in science was a significant positive predictor in Model 1 (β = 0.145, p < 0.001) but became a significant negative predictor in Model 2 (β = −0.256, p < 0.001), reflecting a nuanced relationship with preferences for natural and GE microbiomes. Positive beliefs about science’s ability to improve society were significant in Model 1 (β = 0.127, p < 0.001) and Model 2 (β = 0.258, p < 0.001), showing that this belief was associated with both more preference for natural vs GE microbiomes and more openness to GE microbiomes. This block contributed 1.9% (p < 0.001) of the variance in Model 1% and 15.1% (p < 0.001) in Model 2, highlighting the substantial role of science-specific predispositions in shaping attitudes.

**FIGURE 1:**
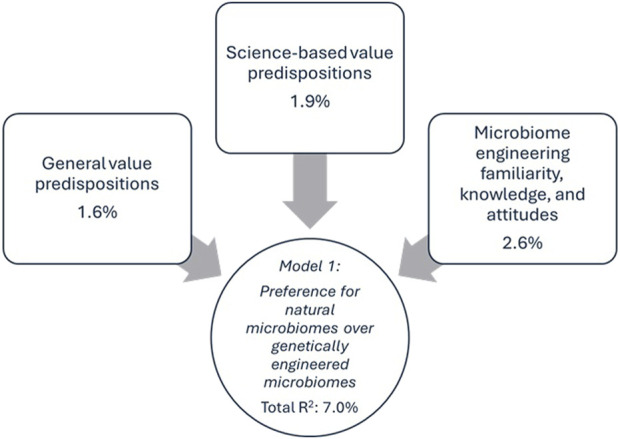
Model 1 influence diagram of significantly influential variables.

**FIGURE 2:**
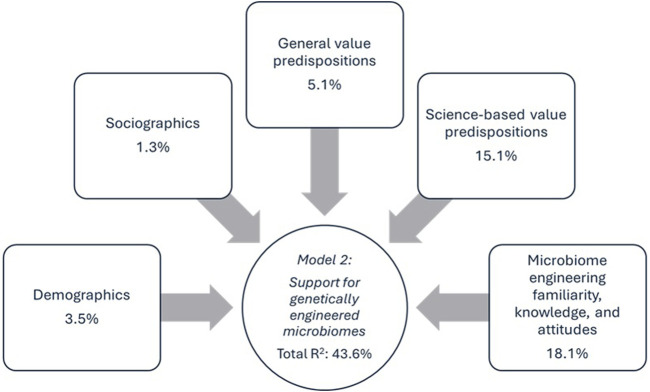
Model 2 influence diagram of significantly influential variables.

The fifth block included familiarity, perceived knowledge, and attitudes toward microbiome engineering. Familiarity with microbiome engineering did not have significant effects in either model. Interestingly, perceived knowledge did not impact preference for natural over GE microbiomes, yet it negatively influenced support for GE microbiomes in Model 2 (β = −0.176, p < 0.001), suggesting that individuals who felt more informed were less likely to support GE microbiomes. Positive attitudes about microbiome engineering’s potential to improve quality of life had the strongest influence in both models (β = 0.212, p < 0.001 in Model 1; β = 0.618, p < 0.001 in Model 2). This block explained 2.6% (p < 0.001) of the variance in Model 1% and 18.1% (p < 0.001) in Model 2, making it the most influential predictor group overall. Finally, it is noteworthy that the total variance explained by Model one was merely 6.9%, compared to 43.6% for Model 2. Table 2 below reports model findings and Figures 1, 2 visually depict the significantly influential variables for each model.

**TABLE 2:** Regression models predicting preference for natural microbiomes over GE microbiomes and support for GE microbiomes.

	Item	Model 1: preference for natural microbiomes over genetically engineered microbiomes		Model 2: support for genetically engineered microbiomes	
		Stand. β coeff.	p-value, sig	Stand.β coeff.	p-value, sig
Block 1: Demographics					
	Age	-0.056	0.101	-0.176	0.000***
	Gender (men = 1; women/nonbinary/transgender = 0)	0.037	0.257	0.030	0.341
	Race (white = 1; nonwhite = 0)	0.061	0.072	-0.031	0.343
	Population Density	-0.019	0.566	-0.002	0.958
	Metro Area Status (metro = 1; nonmetro = 0)	-0.011	0.746	0.051	0.118
	**R** ^**2**^ **(%)**	**0.7%**		4.0%***	
Block 2: Sociographics					
	Highest level of education (1 = four-year college degree; 0 = no college degree)	-0.002	0.944	0.119	0.000***
	Annual income (higher income coded high)	0.028	0.423	-0.028	0.404
	**R** ^**2**^ **Change (%)** **R** ^**2**^ **(%)**	**0.1%** **0.8%**		1.3%** 5.3%***	
Block 3: Value Predispositions—General					
	Political orientation (Republican coded high)	0.024	0.474	-0.232	0.000***
	Church attendance (more frequent coded high)	0.123	0.000***	0.006	0.845
	**R** ^**2**^ **Change (%)** **R** ^**2**^ **(%)**	1.6%*** 2.4%**		5.1%*** 10.4%***	
Block 4: Value Predispositions—Science					
	Distrust in science and scientists	0.145	0.000***	-0.256	0.000***
	Positive belief that science can improve society	0.127	0.000***	0.258	0.000***
	**R** ^**2**^ **Change (%)** **R** ^**2**^ **(%)**	1.9%*** 4.3%***		15.1%*** 25.5%***	
Block 5: Microbiome engineering familiarity, knowledge, and attitudes					
	Familiarity with microbiome engineering	0.030	0.631	0.011	0.824
	Perceived knowledge of microbiome engineering	-0.038	0.551	-0.176	0.000***
	Positive attitude about microbiome engineering’s influence on quality of life	0.212	0.000***	0.618	0.000***
	**R** ^**2**^ **Change (%)** **total R** ^**2**^ **(%)**	2.6%*** 6.9%***		18.1%*** 43.6%***	

p<0.05*, p<0.01**, p<0.001***

Bolded values represent the R^2^ change and total R^2^ values.

## Discussion

This study provides important insights into the factors shaping public preferences for natural microbiomes and support for genetically engineered (GE) microbiomes in the built environment. By employing hierarchical regression models, we identified how demographic, sociographic, and value-based predictors contribute to these preferences, offering a nuanced understanding of the variables that influence attitudes toward microbiome engineering. The findings suggest that while demographic and sociographic factors offer modest explanatory power, value predispositions and topic-specific attitudes play a more prominent role in shaping public perspectives. Results reveal that demographic variables, including gender and race, exert limited direct influence on preferences for natural or GE microbiomes. However, the significance of age in the second model suggests that generational differences may play a role in shaping attitudes, particularly toward emerging technologies like GE microbiomes. Sociographic variables, such as income and education, also provided minimal predictive power, with only education influencing openness to GE microbiomes (i.e., possessing a 4-year college degree was associated with more openness). These findings align with previous research indicating that demographic and sociographic factors often serve as baseline influences, contextualized by broader value systems and beliefs (Cummings, Landreville and Kuzma, 2024; Critchley, 2018).

General value predispositions, such as political orientation and religious engagement, contributed more notably to the models. While political orientation did not impact preference for natural microbiomes, it did impact openness to GE microbiomes, with Democratic Party orientation being more open. Our finding reflects past research that has found that left-wing political orientations are more likely to support biotechnology progress (e.g., Critchley, 2018). The significance of church attendance in the first model highlights the role of cultural and moral frameworks in shaping preferences for natural microbiomes. However, its diminished significance in the second model suggests that these general predispositions may be less influential when specific attitudes about GE microbiome engineering are considered. This underscores the importance of accounting for both general and topic-specific values in understanding public attitudes.

Science-specific value predispositions emerged as significant predictors, with a contrasting effect between the models with distrust. Distrust in science positively predicted preferences for natural microbiomes in the first model but negatively predicted support for GE microbiomes in the second. These findings highlight the complex interplay between trust in scientific institutions and public attitudes toward biotechnology. Positive belief about science’s societal benefits was also influential. Optimistic views of science yielded both preference for the natural and openness to GE microbiomes. In other words, it appears that positive beliefs about science improving society can encourage preference for natural microbiomes over GE microbiomes but still encourage openness to GE microbiomes.

Positive attitudes about microbiome engineering’s potential to improve quality of life had the strongest influence across both models, indicating that perceived benefits play a central role in shaping support for these technologies. Interestingly, perceived knowledge negatively predicted support for GE microbiomes in the second model, suggesting that individuals who believe they are more informed may also perceive greater risks or hold more critical views of GE applications. These findings emphasize the need for nuanced communication strategies that address not only public knowledge gaps but also the underlying attitudes that drive perceptions.

Overall, the models highlight the multifaceted nature of public attitudes toward microbiome engineering in the built environment. While demographic and sociographic factors provide a foundation, value predispositions and specific attitudes about microbiome engineering exert the greatest influence. These findings suggest that future research and engagement efforts should focus on addressing both general trust in science and the specific concerns and benefits perceived by the public regarding microbiome engineering technologies. Below, we further explicate how interested groups can use these findings to stay attuned to these complexities and better align technological development with diverse public perspectives.

### Model comparison

The substantial difference in explained variance between Model 1 (7.0%) and Model 2 (43.6%) underscores the nuanced and multifaceted nature of public preferences for microbiome engineering, particularly the strong preference for non-GE microbiomes among certain audiences. Indeed, there are very few respondents who *did not* prefer natural microbiomes (i.e., merely 8.4%). Because the majority of respondents expressed preference for natural microbiomes over GE microbiomes (61.5%) or expressed uncertainty (30.1%), there was less variance available to explain in the dependent variable compared to the support for GE microbiomes dependent variable (Model 2), where respondents differed more in their opinions. In Model 2, 16.6% of respondents were *not* open to GE microbiomes, 29.3% were unsure, and 54.1% were open. In other words, the lack of variance in the dependent variable of preference for natural microbiomes (Model 1) may be driving the low total variance explained by Model 1; most people prefer the natural. Alternatively, the set of independent variables that were included in Model 1 may not be the most relevant and appropriate variables to include in the model to predict preference for natural microbiomes. However, this explanation is less likely when we consider that Model 2 has such a high total variance explained, uses the same independent variables, and is focused on a very similar topic in microbiome engineering. Additionally, the dependent variables are not correlated (*r* = 0.02, *p* = 0.407). In short, most respondents prefer natural over GE microbiomes; however, just because a respondent *prefers* the natural, that does not mean that same respondent *rejects* GE microbiomes if their benefits are well-documented and their risks are considered.

Another important finding is the similar model results in terms of the predictive significance of topic-specific attitudes as compared to general demographic and sociographic factors. While demographics and sociographics provide baseline context, the stronger influence of attitudes, such as positive perceptions of microbiome engineering’s potential benefits, suggests that public preferences are primarily driven by how individuals understand and evaluate the technology’s implications rather than by their broader characteristics. This indicates that attitudes toward microbiome engineering are more dynamic and closely tied to the perceived relevance and value of the technology.

Interestingly, variables including age, political orientation, distrust in science and scientists, and perceived knowledge all demonstrated negative relationships in Model 2, suggesting that skepticism about GE applications is deeply rooted in both demographic and attitudinal factors. Older individuals and people with political orientation toward the Republican Party, for instance, may hold more traditional views or exhibit greater caution toward emerging biotechnologies, contributing to less openness to GE microbiomes. Similarly, distrust in science likely reflects broader concerns about the motivations and reliability of scientific institutions, amplifying opposition to GE innovations. Perceived knowledge also negatively influenced support for GE microbiomes, which may seem counterintuitive but highlights the complexity of public perceptions. Individuals who believe they are well-informed may have developed a critical stance or harbor heightened concerns about risks associated with genetic engineering. This finding underscores the need for engagement strategies that not only provide information but also address potential pre-existing skepticism and misconceptions. In contrast, positive attitudes about microbiome engineering’s potential to improve quality of life were strongly and positively related to support for GE microbiomes. This variable emerged as the most significant predictor of Model 2, indicating that perceptions of societal and individual benefits are crucial in shaping acceptance. These findings suggest a divide in public preferences, with some audiences skeptical of GE microbiomes while others are more open to GE applications if they perceive tangible benefits.

### Tailored engagement strategies for different audiences: aligning with Responsible Research and Innovation

The results from both regression models underscore the critical need for adopting tailored engagement and communication strategies to foster more robust and meaningful public engagement around microbiome engineering. A one-size-fits-all approach will not effectively resonate with the diverse perspectives held by different segments of the population. Instead, interested parties should adopt a segmented communication strategy that addresses the specific concerns, values, and characteristics of various groups that will facilitate informed and productive discussions about the potential benefits and risks of microbiome engineering, fostering deeper, more reflective engagement. The insights from the models suggest that interested groups should move beyond the goal of securing immediate approval or consensus from the public. Instead, the focus should be on creating open, honest, and thoughtful conversations tailored to the distinct perspectives of different demographic groups. By doing so, respect for the diverse viewpoints that exist within society can be better maintained, which invites a more inclusive and participatory form of dialogue. Such a strategy not only enhances the quality of public discourse but also deepens the level of public understanding of complex scientific advancements, allowing individuals and communities to engage with the potential long-term impacts of microbiome engineering.

This approach aligns closely with the principles of RRI, which call for inclusivity, transparency, and responsiveness in the development and deployment of microbiome engineering. RRI emphasizes the importance of actively involving the public and considering their values and ethical concerns throughout the research and innovation process. By incorporating these principles, partners ensure that microbiome engineering is not only innovative but also ethically grounded and socially responsive. The public becomes a key participant in shaping how microbiome technologies are understood, evaluated, and ultimately integrated into society, rather than passive recipients of pre-determined outcomes.

For example, when engaging with religious communities, the communication and engagement should be sensitive to traditional values and ethical concerns. These groups may view certain technological advances through a moral or spiritual lens, which makes it crucial to frame the conversation around compatibility with their beliefs rather than attempting to overcome perceived opposition. Engaging trusted community leaders and facilitating open discussions about the ethical implications of microbiome engineering could foster more thoughtful dialogue about its potential role in society. By creating space for reflective consideration, partners encourage these communities to actively participate in shaping the direction of the technology, a key component of RRI. This approach promotes inclusivity and responsiveness, ensuring that technological development is not isolated from the moral and cultural frameworks that guide various communities.

Outreach efforts targeting younger, more educated, and politically progressive audiences could focus on the broader societal implications of the technology. These groups are often driven by a desire to solve global challenges such as climate change, public health crises, and inequality. They may already prioritize health-conscious, sustainable, or environmentally focused practices, so messaging that emphasizes how the technology supports these ideals will likely foster deeper engagement. Instead of attempting to gain favor, partners could highlight how the innovation aligns with these communities' broader goals of wellbeing, environmental stewardship, or personal safety. Framing conversation around how innovation contributes to long-term solutions, promotes sustainability, or advances social equity will likely engage these groups more deeply. In doing so, technology developers align the discourse with RRI’s aim of anticipating the broader impacts of technological innovation, ensuring that societal needs and challenges are addressed in research and development processes. By focusing on societal impact, these efforts encourage responsible innovation that seeks to improve public welfare while minimizing risks.

When addressing communities with high levels of distrust in science or institutions, the communication strategy could prioritize transparency, open dialogue, and active listening. These individuals may be skeptical of technological advancements due to concerns about the motives of scientists, corporations, or policymakers. To foster meaningful engagement, it is crucial to provide clear, transparent information about both the potential benefits and risks of the technology, while allowing space for questions, concerns, and critique. Public forums, consultations, and interactive discussions could help break down barriers of mistrust and build a more collaborative dialogue. This approach directly aligns with RRI’s commitment to transparency and co-creation, where the public is invited to engage actively in the decision-making process, contributing their views on the ethical and societal aspects of new technologies. By encouraging two-way communication and shared responsibility, technology developers ensure that the innovation process is more democratic and reflective of diverse perspectives.

It is also of paramount importance to note that transparency and accountability are key across all audience segments. Providing access to comprehensive information about the development, regulation, and oversight of new technologies could empower individuals to make more informed judgments about the risks and benefits. Additionally, highlighting ethical considerations and how potential risks are managed—through safety testing, regulatory processes, or long-term monitoring—could deepen the conversation about innovation. RRI emphasizes the importance of accountability throughout the research process, ensuring that technologies are developed with public values and ethical considerations in mind. By being upfront about the limitations and uncertainties of new technologies, technology developers encourage a more thoughtful and balanced discourse, enabling audiences to critically engage with both the positive and negative aspects of the technology.

## Conclusion

The findings from this study provide valuable insights into the factors that shape public attitudes toward microbiomes, both natural and genetically engineered. By identifying the key demographic, sociographic, and attitudinal predictors of microbiome preferences, technology developers and partners can deliver more targeted and effective communication strategies that resonate with the values and concerns of specific communities. Tailoring engagement efforts to the unique characteristics of different groups, while addressing broader concerns about scientific trust and transparency, will be critical for fostering greater public acceptance of microbiome-related innovations. Ultimately, a nuanced understanding of these predictors will enable policymakers, scientists, and companies to engage in more informed, inclusive, and productive dialogues about the future of microbiome engineering. Given the increasing societal relevance of microbiome technologies, these findings offer timely insights for aligning emerging innovations with public values and policy priorities.

## Data Availability

The datasets presented in this article are not readily available because Under data supervision of the research team. Requests to access the datasets should be directed to clcummin@ncsu.edu.
