# Supplementary material for: Natural vs. genetically engineered microbiomes: understanding public attitudes for indoor applications and pathways for future engagement
Source: Front Genet. 2025 Mar 26;16:1560601. doi: 10.3389/fgene.2025.1560601 (PMC11979166; doi:10.3389/fgene.2025.1560601)
Supplement: Supplementary file 1 [file Supplementaryfile1.docx]

Natural vs. Genetically Engineered Microbiomes: Understanding Public Attitudes for Indoor Applications and Pathways for Future Engagement

**Supplementary Materials**

The full list of items used to create the independent variable scales are provided below.

**Distrust in science/scientists**

1. Scientists don't value my concerns when making decisions.
2. Scientists purposefully hide the truth from the public.
3. Using science isn't the best way to find answers to the biggest questions of society today.
4. Scientists think they are more important than everyone else.

**Science improves society**

1. Our leaders should use technology to solve problems in society.
2. We should use technology to improve our daily life.
3. Science is vital to the success of our society.

**Familiarity with microbiome engineering**

1. I am familiar with the concept of microbiome engineering.
2. I am aware of the different techniques and technologies used in microbiome engineering.
3. I feel well-informed about how microbiome engineering can impact air quality and health in indoor spaces.

**Perceived knowledge of microbiome engineering**

1. I have a basic understanding of how microbiome engineering can be applied.
2. I feel confident in my knowledge of the potential benefits of microbiome engineering in enhancing the built environment.
3. I believe I have a good grasp of the potential risks associated with microbiome engineering.
4. I feel knowledgeable about the current research and developments in microbiome engineering for improving indoor spaces.

**Positive attitudes toward microbiome engineering**

1. I believe that microbiome engineering can play a significant role in addressing environmental challenges.
2. I have a positive attitude toward the concept of microbiome engineering.
3. I believe that microbiome engineering can significantly improve the quality of indoor spaces.
4. I find the idea of using microbiome engineering to enhance the built environment appealing.
5. I am enthusiastic about the potential benefits of microbiome engineering for indoor air quality.
6. I have a favorable opinion of organizations and initiatives that promote microbiome engineering.
7. I think microbiome engineering is an innovative and promising approach for sustainable architecture.
8. I would support the implementation of microbiome engineering in public spaces.
9. I believe that microbiome engineering can contribute to a healthier and more comfortable living environment.
10. I would be open to using products or services that incorporate microbiome engineering principles in my home or workplace.
